# A Biogeographical Debate at the Origins of Limnology in Switzerland and Italy: The Issue over Pelagic Fauna Between Pietro Pavesi and François-Alphonse Forel

**DOI:** 10.1007/s10739-024-09791-7

**Published:** 2024-10-16

**Authors:** Pier Luigi Pireddu

**Affiliations:** Centro Interuniversitário de História das Ciências e Tecnologia (CIUHCT), Faculdade de Ciências (FCUL), Universidade de Lisboa, Campo Grande C4, Lisbon, Portugal

**Keywords:** History of limnology, History of ecology, Limnology in Italy, François Forel, Pietro Pavesi, Pelagic fauna

## Abstract

This article explores the early biogeographical debates that shaped the beginning of limnology, focusing on the differences of opinion concerning the origins of pelagic fauna between two pioneering scientists: Pietro Pavesi and François-Alphonse Forel. The study examines how Pavesi’s hypothesis of a marine origin for pelagic fauna contrasts with Forel’s theory of passive distribution, situating their arguments within a broader Darwinian framework. The first part of the paper provides a historical overview of Italian limnology, highlighting Pavesi’s contributions and interpreting Forel’s writings to underscore the significance of discovering pelagic fauna in conceptualizing lakes as microcosms. The second part compares Pavesi’s and Forel’s hypotheses, emphasizing their impact on the scientific understanding of freshwater ecosystems. The importance of this discovery, in both historical and scientific contexts, lies in recognizing the presence of plankton in lakes as a crucial element for the mature formulation of ecological concepts, such as the ecosystem.

## Introduction

Between 1860 and 1880, the research field of limnology emerged in Europe and the United States. In the North American context Stephen Forbes, who coined the term “microcosm” to refer to limnological ecosystems (Forbes 1887), is considered its founder (McIntosh 1980, 1985; Hagen 1992; Golley 1993; Schneider 2000). The dynamics behind the establishment of limnology in the United States and its theoretical implications have been discussed extensively (Elster 1974; Cooper 2003; Egerton 2014, 2016; Dussault 2024). In Europe, François-Alphonse Forel (1841–1912) played a central role in establishing the discipline with his research on Lake Geneva and Lake Léman in the 1870s and 1880s. This essay focuses on the discovery of the small pelagic fauna (zooplankton) in lake systems, in which European research, particularly on lakes in Switzerland, Scandinavia, and northern Italy, figured prominently. A consequence of this discovery was the triggering of several scientific disputes that played a key role in the formation of limnology as a new scientific discipline. Here I describe one such dispute, between Forel and Pietro Pavesi (1844–1907), on the origin of the small pelagic fauna in freshwater systems that illustrates the role that Darwinian thinking came to play in early limnology.

The term small pelagic fauna describes a community of typically microscopic organisms belonging to various systematic groups, ranging from protozoa to crustaceans. Except for a few species that possess rudimentary locomotion devices, these plankton cannot counteract the currents’ motions (Bertoni 2018). Therefore, plankton are carried by the body of water they inhabit, mirroring their movements in the lake. Borrowing the term introduced by Rina Stella Monti (1871–1937), these organisms can be viewed as a floating society – *società fluttuante*.

This essay explores the discovery of floating societies in freshwater ecosystems through two main topics. I first describe the role this discovery played in the development of limnology, and, drawing on Forel’s writings from 1888, I propose an interpretation largely overlooked in recent analyses of his scientific contributions. Second, I examine the controversies around the origin of these organisms and attempt to clarify the debate’s significance in scientific-historical context. In their writings on freshwater ecosystems, Pavesi and Forel offered two different hypotheses to explain the phylogeny of the small pelagic fauna. Pavesi ascribed marine origins to them, the species physiologically transiting from marine to lacustrine environments, while Forel defended a passive mode of distribution of new species (modified for freshwater survival) from freshwater centers of origin. I argue that this debate should be understood as a biogeographic-limnological problem that was resolved within a Darwinian evolutionary framework. This perspective helps fit the history of limnology into the general history of ecology and history of biogeography, where it has been largely neglected (Bueno-Hernández et al. 2023). Furthermore, the debate illustrates central themes that characterized the emergence of limnology as a scientific discipline. These can be summarized into four groups: the impact of the Ice Age;^1^ the discovery of zooplankton as nutrient reservoirs and trophic levels more generally; biogeographical issues concerning the distribution and diffusion of freshwater microorganisms; and Darwinian ideas about adaptation through natural selection. These themes converge in the debate between Pavesi and Forel, highlighting assumptions such as a connection between current lake ecology and biogeographical history characteristic of limnology.

The essay is organized in two main parts. The first gives a historical overview of the Italian limnological scientific context of the late 19th century. It then moves to the figures of Pavesi and Forel, describing their biographies and their scientific writings, and discusses the discovery of zooplankton and its place in the history of limnology. The second part delves into the debate on the origin of the zooplankton in a broader international context. There it considers Forel’s and Pavesi’s hypotheses, comparing their views and highlighting points of convergence and divergence, and discusses some implications of the debate. In doing so it contributes to the literature on Pavesi’s contributions to limnology, which remain understudied in contrast to extensive literature on Forel’s scientific achievements (Egerton 1962; Campanella 2016; Berg 1950). Also, it addresses broader issues such as the development of an ecological perspective in the study of lakes and in biology generally in the late 19th century, and the transnational nature of environmental science (Casado and Montes 1992; Casado 2011).

## The Emergence of Limnology in Italy

The earliest studies on Italian lake systems appeared in the 17th century. Athanasius Kircher (1665) published the first works examining lake Albano and lake Nemi. During the 18th century, Ferdinando Marsili (2004), provided an in-depth study on Lake Garda, the largest Italian lake.^2^ In the first half of the 19th century, several scientists took an interest in the study of lakes: Procaccini Ricci and Bartolomeo Borghi dedicated themselves to the study of lakes across the peninsula (Ricci 1814; Borghi 1821). These works were pioneering individual attempts to understand the natural mechanisms of lake systems; they did not, however, form part of a coordinated research program. The limnological studies conducted before the second half of the 19th century could be defined as proto-limnological (McIntosh 1985).

The second half of the 19th century constituted a turning point in this area of research, with the noteworthy works of Giovanni Battista Maggi on Lake Maggiore, and Emilio Spreafico and Bartolomeo Gastaldi on the lakes of Piedmont (Maggi 1857; Gastaldi 1863, 1865; Spreafico 1879). Olinto Marinelli, Adriano Garbini, Leopoldo Maggi, and Pietro Pavesi emerged as the first Italian scientists to undertake a methodical study of lakes. They specialized differently: Marinelli studied the lakes of the Veneto, focusing on geophysical issues, while Maggi, Garbini, and Pavesi, focusing on limnobiological issues, made significant contributions to knowledge of microorganisms. During this early period, two limnological domains coalesced: the geographical (concerning morphometry and the study of lake genesis and water physics), and the biological.^3^ The boundary between them was, however, permeable – early research in the geographical field often addressed biological issues, and vice-versa. Debates from this period, such as those concerning the small pelagic fauna, often involved experts from both domains.

**Fig. 1:**
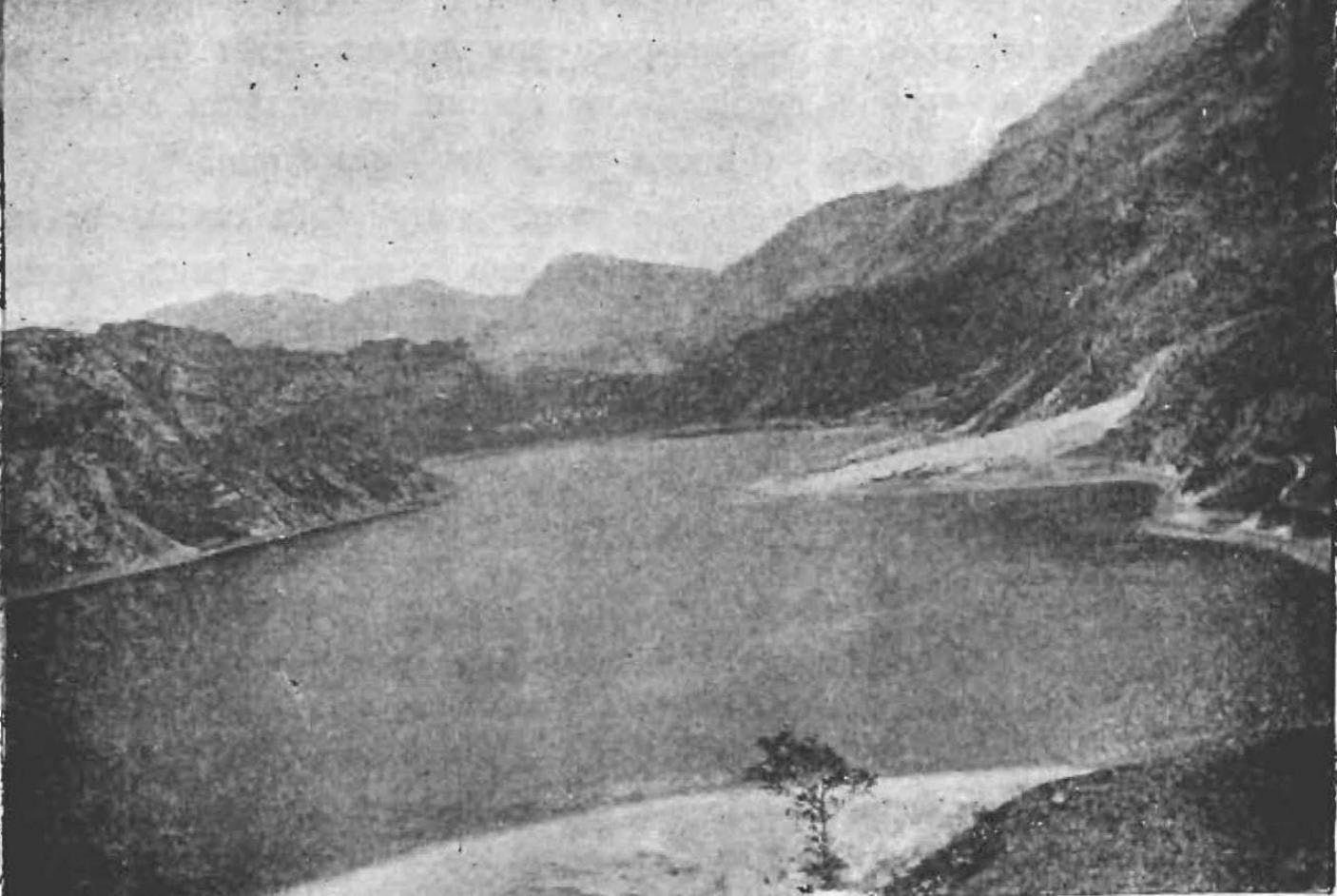
Picture of Lake Cavazzo, in the Italian region of Friuli (Marinelli 1894a, p. 179)

Marinelli and Antonio Stoppani were leading figures in geographically oriented research.^4^ Marinelli collected data on 115 lakes, culminating in an original work on the essential characteristics of these lacustrine systems: location, height above sea level, surface area, and maximum depth data (Marinelli 1895). Building on Forel’s research (described below), he published one of the first attempts to classify Italian lakes, and undertook in-depth research on Lake Cavazzo (Fig. 1). In this work Marinelli focused chiefly on morphology and topography (Marinelli 1894a, b). He provided data on various physical parameters, including water properties such as temperature, color, and transparency, and geochemical factors such as past glaciation, climate, and wind patterns. Notably, his work sidestepped biological discussions and concentrated instead on the lake’s origin.

Earlier, Antonio Stoppani had attempted to clarify the origin of lake systems, turning his attention to the geological impact of the Ice Age in Northern Italy (Stoppani 1879). He reviewed existing theories on lake origins and proposed that Alpine lakes were once sea branches or fjords filled by advancing glaciers.^5^ This inference is in line with Pavesi’s hypothesis on the marine origin of the small pelagic fauna (Stoppani 1881). Indeed, Stoppani argued that Pavesi’s work supported his own theory on the geological origins of lakes.^6^

**Fig. 2:**
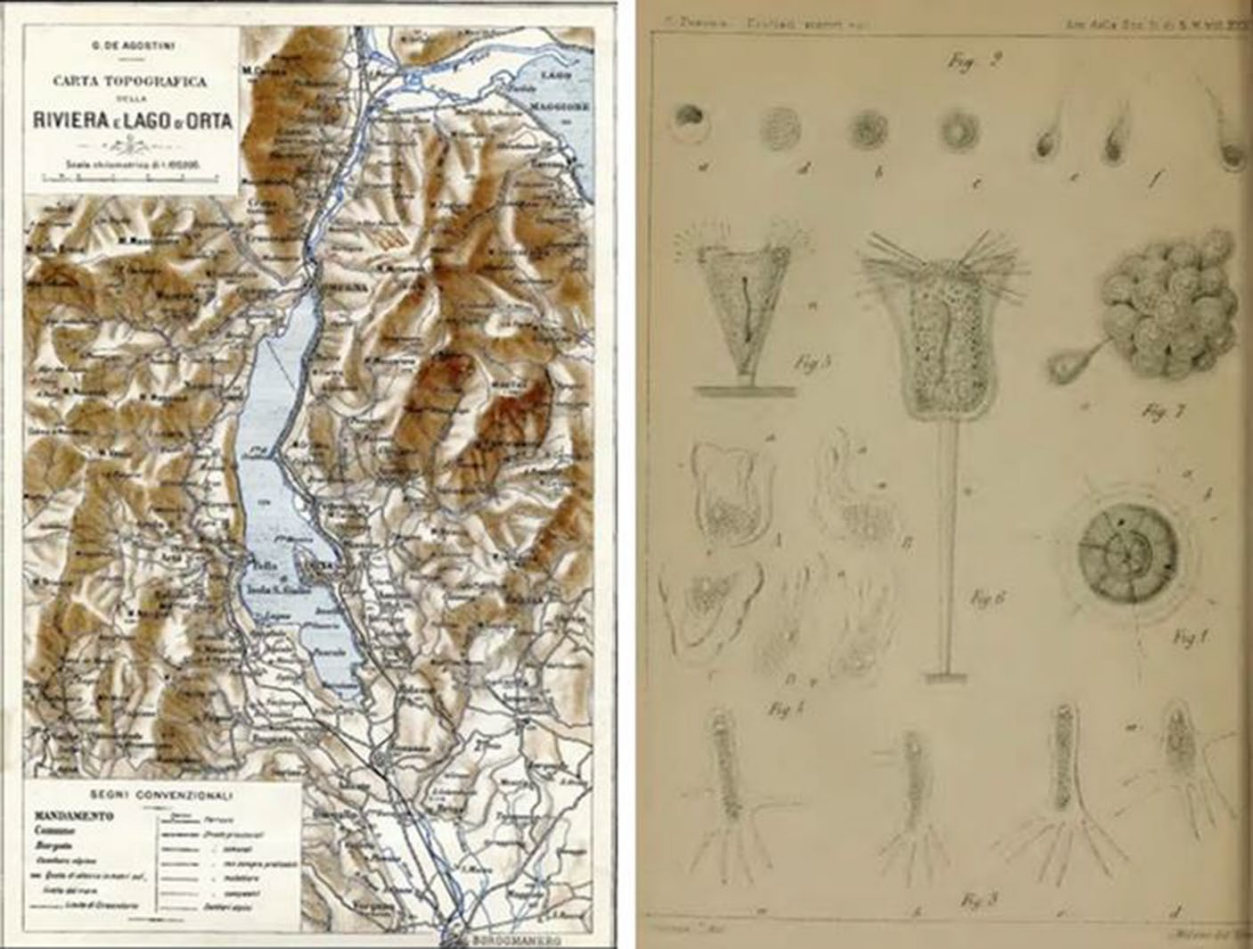
Left: Topographical chart of Lake Orta (De Agostini 1897). Right: protists of Sardinia (Parona 1883)

Several early limnologists, including Leopoldo Maggi, Carlo Cattaneo, and Corrado Parona advanced the biological approach through studying microorganisms in Italian lakes from the late 1870s. Such studies on protozoa garnered both national and international attention, with scholars such as August Gruber contributing to the field (Gruber 1880). Cattaneo pursued studies of microfauna, extending his work to the protists of Lake Como (Cattaneo 1878, 1882). Similarly, Norsa was the first to study the microorganisms in the waters of the Mantua region, particularly Lake Superior (Lago Superiore) (Norsa 1879, 1881). The earliest research on microorganisms inhabiting Lake Orta was conducted by Parona in 1880 (Parona 1880a, b) (Fig. 2). He concentrated on protozoa, with a special emphasis on the class of rhizopods. From 1881 to 1883, Parona also spent three years in Sardinia exploring the island’s fauna, particularly microfauna and protists, publishing some of his work in international journals (Parona, 1882a, b, 1883).

Leopoldo Maggi was another key figure in the early Italian limnological tradition (Pavesi 1905).^7^ In the 1880s he championed the scientific value of analyzing protists and protozoa, both in biological and limnological perspectives (Maggi 1884). He concentrated on the classification of protists (Maggi 1881a, b), and provided an account of Lake Loppio (Maggi 1881c). He also published a textbook on protists and was the first to offer a university course on the topic (Maggi 1882). Maggi rooted his research in Pavesi’s writings: “The primary objective of my investigation was to determine the types of protists present in the waters of our lakes; however, after the research of the esteemed Prof. Pietro Pavesi on their pelagic fauna, this examination also appeared interesting from that perspective” (Maggi 1881a, p. 18, trans. author).^8^

The question of pelagic fauna in freshwater ecosystems entered the Italian limnological debate very early and drew in scholars from a variety of disciplines. The activity of Pavesi was crucial in spurring this interest in lake zooplankton — the floating societies.

## Pietro Pavesi (1844–1907): Biographical Notes and Limnological Research

Pietro Pavesi was an Italian arachnologist, limnologist, ichthyologist, ornithologist, and zoologist, who today is recognized as the founder of Italian limnology, and one of the pioneers of lacustrine biology worldwide (Bonomi 1907; Monti 1909). He was born in Pavia in 1844, graduated from the University of Pavia in 1865, and became a professor at the same university in 1875. On October 26, 1877, Pavesi wrote a letter to Guelfo Cavanna documenting the first discovery of the small pelagic fauna in Italian lakes including copepod and cladoceran crustaceans, such as *Leptodora hyalina*, *Heterocope robusta*,* Bythotrephes longimanus*, and *Daphnia* species. His initial research revealed several key points: that Italian small pelagic fauna resembled those found in other lakes in Europe and the United States; most of the species recorded were new; and Italian lake fauna displayed lower species diversity than other regions that had been surveyed, such as Scandinavia. A few years later, Pavesi revisited the topic and significantly expanded his research. His main purpose was to understand the distribution patterns of these microorganisms across Italian lakes. This required analyzing a substantial number of lakes to accurately map their species distribution. He also investigated factors influencing their distribution, including depth requirements, bathymetric limits, surface conditions, water clarity, and temperature (Pavesi 1879a, b).

**Fig. 3:**
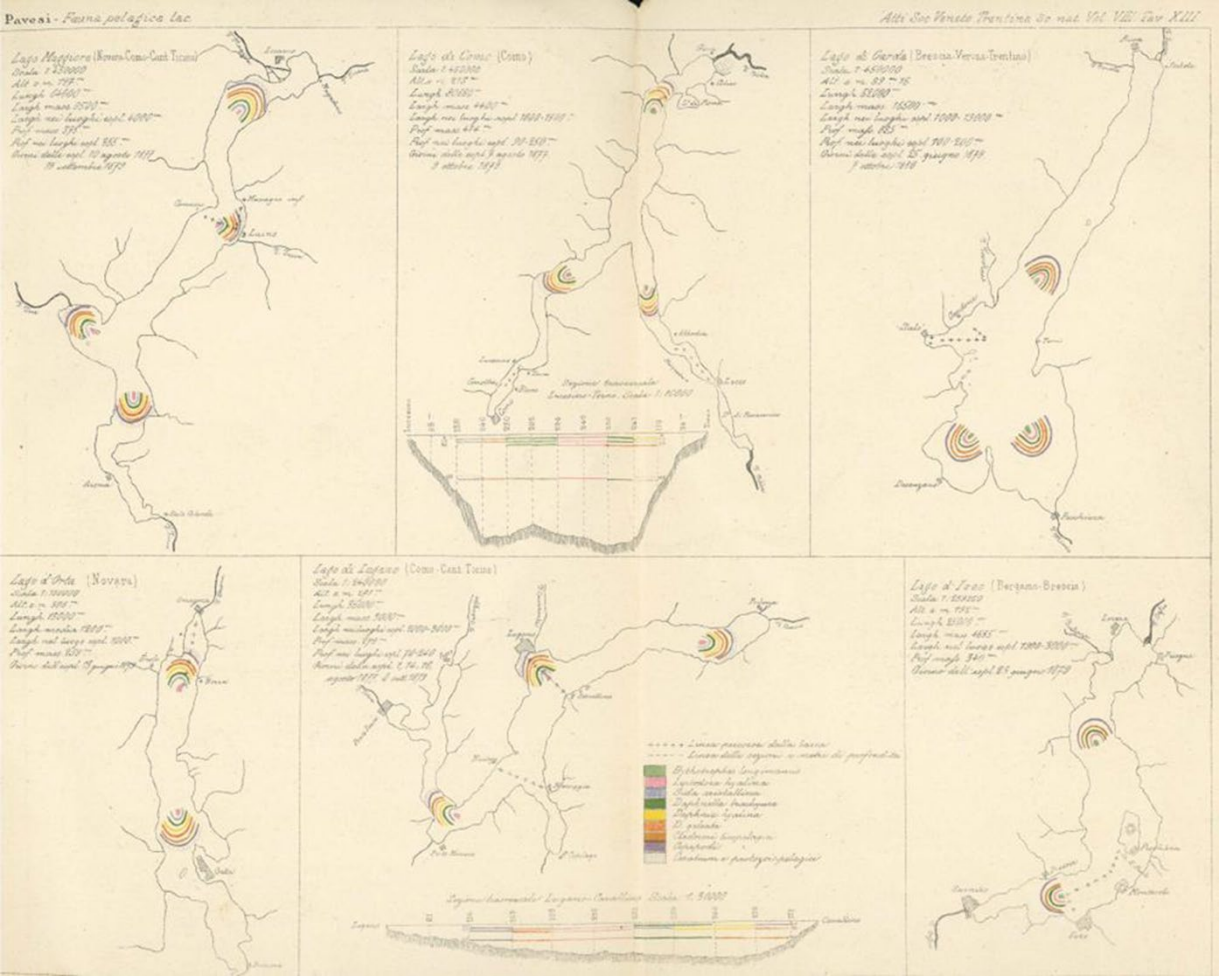
Maps showing the lakes where pelagic fauna research has been conducted. In this chart, only six of them are indicated: Maggiore, Como, Garda, Orta, Lugano, and Iseo (Pavesi 1883)

**Fig. 4:**
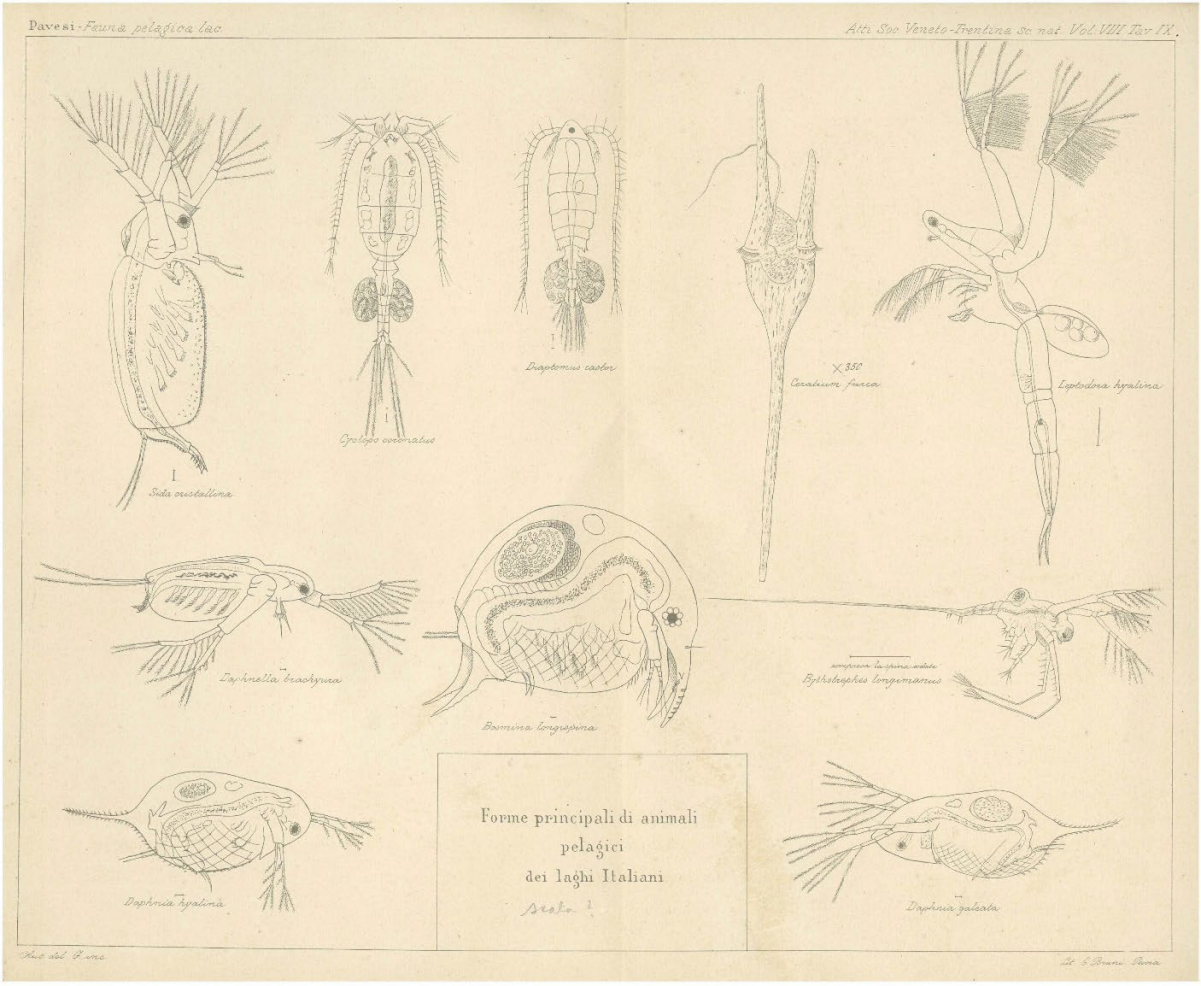
The most common pelagic fauna in Italian lakes (Pavesi 1883)

In his most comprehensive work on pelagic fauna, Pavesi examined Alpine lakes across Italy (Pavesi 1883). He provided a scientific analysis of the small pelagic fauna of each lake, highlighting the characteristic species and their distribution throughout the region (Fig. 3).^9^ In this study, he argued for the marine origin of these microorganisms. Additionally, he provided a detailed account of the most common species, emphasizing the significance of entomostracans as a core component (Fig. 4). In the same period Pavesi also published two articles in French on the topic of the small pelagic fauna, the only non-Italian works in his bibliography (Pavesi 1880, 1889). His discussion of three lakes in the Ticino region – Muzzano, Piano, and Delio – is particularly noteworthy (Pavesi 1889).

Pavesi’s pioneering work in limnology, encompassing both major publications and minor contributions, can thus be summarized in a few key points. First, Pavesi was the first to document the small pelagic fauna in the Canton of Ticino and various lakes across Italy systematically. This research significantly expanded the understanding of Italian freshwater ecosystems, adding numerous species and even new genera to the country’s known fauna. Second, Pavesi’s meticulous observations provided valuable insights into the life cycles of pelagic crustaceans, including their daily activity patterns, preferred habitats, and migratory behaviors. Third, he proposed a novel hypothesis suggesting the marine origin of the small pelagic fauna, to be discussed below.

## François-Alphonse Forel (1841–1912): Biographical Notes and the Foundation of a Discipline

The first significant investigations of Swiss lakes were conducted by Francois Forel. Born in Morges, Switzerland, Forel studied at the Académie de Genève, and attended medical school in Montpellier and then the University of Würzburg, where he completed his education with a dissertation on muscle anatomy and physiology. Early in his career, Forel became actively engaged in limnological research. Forel’s contributions to limnology began with the work *Introduction à l’étude de la faune profunde du Lac Léman* (1869), when he described the so-called abyssal fauna at the lake bottom.

Extensive literature is available on Forel. On the centenary of his death in 2012, the journal *Archives des Sciences* published a special volume devoted to Forel’s life and historical impact. This issue explored Forel’s biography, scientific contributions, and the ongoing work of the Forel Institute – today the Department F. A. Forel for Environmental and Aquatic Sciences. Vincent and Bertola contextualized Forel’s scientific activity and his establishment of limnology as the “oceanography of lakes.”^10^ Earlier, Berg emphasized Forel’s foundational role in the field, noting his early focus on plankton research (Berg 1950). Egerton credited Forel with the discovery of small pelagic fauna and the pioneering division of lakes into three zones: littoral, pelagic, and bottom. Egerton also hinted at Forel’s broader theories on the origins of these lake species (Egerton 1962).^11^ More recently, Vincent and Bertola (2014a, b) have suggested that Forel’s lesser-known contributions include the concept of the lake as a microcosm (see below) and the nature of the pelagic and littoral fauna (on deep fauna, Campanella [2016] has also given a recent account). Similarly, Bertola (1998) considered that Forel’s account of Lake Leman was one of the first scientific descriptions of an ecosystem, and that his use of the lake as an ecological model was in line with Forbes (1887).^12^

However, Forel’s research on small pelagic fauna and its role in the development of limnology has received little attention in the proposed literature. Bertola discusses the concept of the lake as an “ecological model” and draws a parallel between Forbes and Forel regarding the idea of the lake as a microcosm (Bertola 1998). Nonetheless, Bertola does not explore the significance of the discovery of a new trophic level, zooplankton. I argue that for Forel, pelagic fauna represented the missing link in the food chain, providing a more complete understanding of the organic matter cycle and solidifying the microcosm concept of the lake (Forbes 1887). Forel discussed this issue both in his major (Forel 1885, 1892–1904) and his less-known writings (Forel 1876a, 1877b, 1880, 1882b, 1888).^13^

**Fig. 5:**
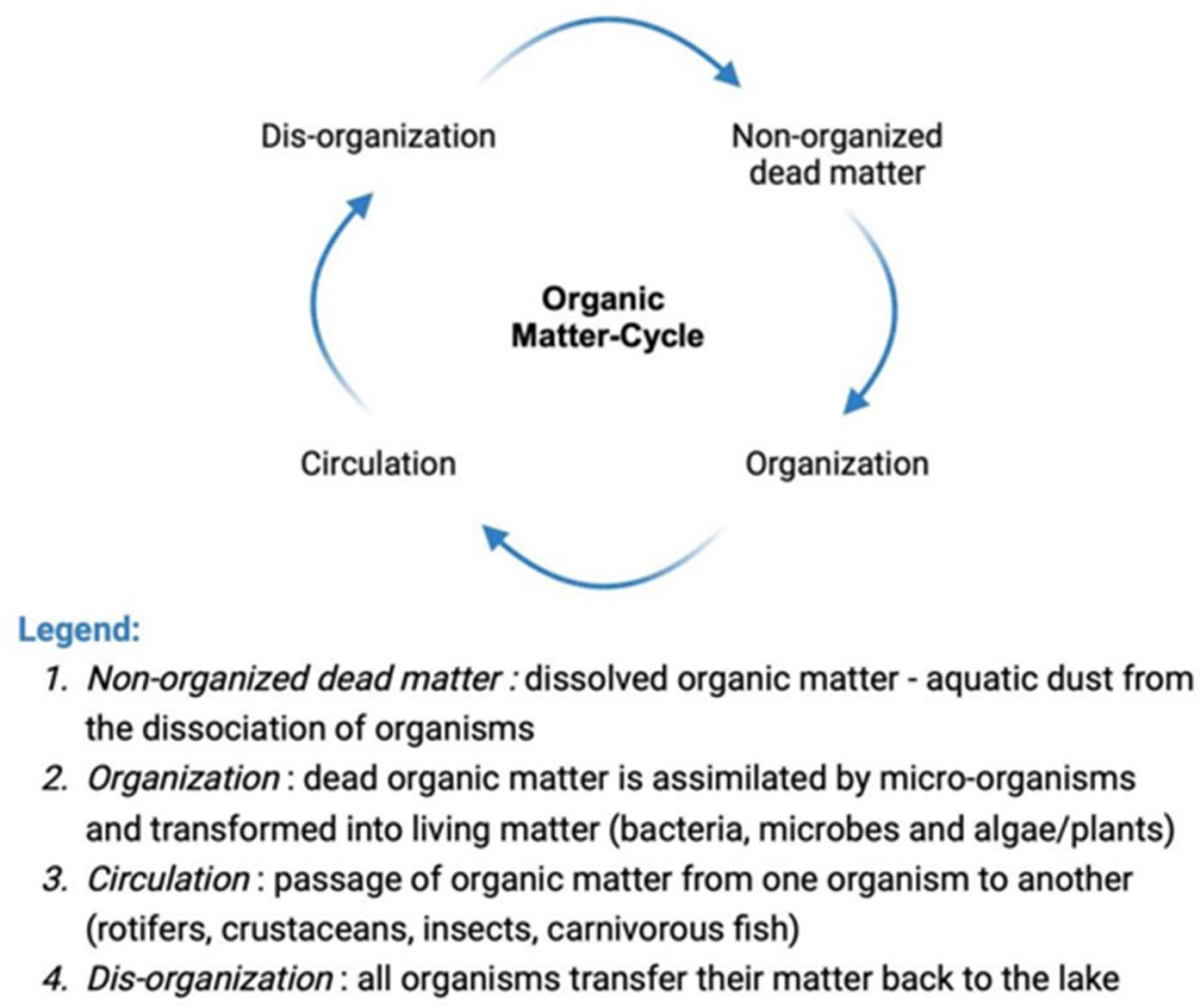
Diagram elaborated from Forel (1888)

Forel demonstrated that the vertical zones of a lake are interconnected blocks, rather than three distinct parts with separate internal dynamics. Abyssal fauna are linked to the fauna elsewhere, such as the pelagic zone, because they feed on each other. Ultimately, the lake is a single unit composed of three integrated zones. From a limnological point of view and, even more broadly, from an ecological point of view, this discussion is significant as it highlights the dynamic interrelations of the various components of the lake, where natural processes involve the entire system. Thus for Forel, the discovery of small pelagic fauna (zooplankton) provided the *missing links* to treat the lake as an entire unit. In a brief 1888 account – *Les micro-organismes pélagiques des lacs de la région subalpine* – Forel discussed the trophic significance of small pelagic fauna in detail. He asserted that “[t]hese discoveries are precious, they give us the missing links that we lack for understanding the cycle of the circulation of organic matter in our lakes, between dead and living nature (Forel 1888, p. 170, trans. author).^14^ The cycle of organic matter to which Forel (1888) referred consisted of four essential stages (Fig. 5). This cycling concept represents a major achievement resulting from the discovery of the small pelagic fauna in lake systems.

Thus, limnologists came to think about the dynamics of life in lakes as cyclical, just as they had come to view geochemical and (to some extent) geological change as cyclical (Gorham 1991). Indeed, the organisms that inhabit different lake areas are interconnected in terms of biotic and abiotic interactions. The discovery of the presence of plankton in lakes (small pelagic fauna), together with other organisms in sediments, should be regarded not only as a significant step in the direction of identifying mutual interactions between organisms, such as trophic interactions and the occupation of ecological niches, but also between organisms and the environment (water, catchment area, etc.). These are prerequisites for understanding the development of the contemporary notion of ecosystem.^15^

## The Emergence of the Small Pelagic Fauna as a Research Topic

In the second half of the 19th century, the discovery of pelagic fauna engaged numerous experts worldwide, and led to the formulation of scientific questions regarding the origin and distribution criteria of pelagic species. The scientific problems to be addressed included questions such as: How do the planktonic species settle in a territory? What is their homeland of origin? This line of questioning forms the beginning of *limnological biogeography*, characteristic of limnology in the last decades of the 1800s (Baldi 1950).

**Fig. 6:**
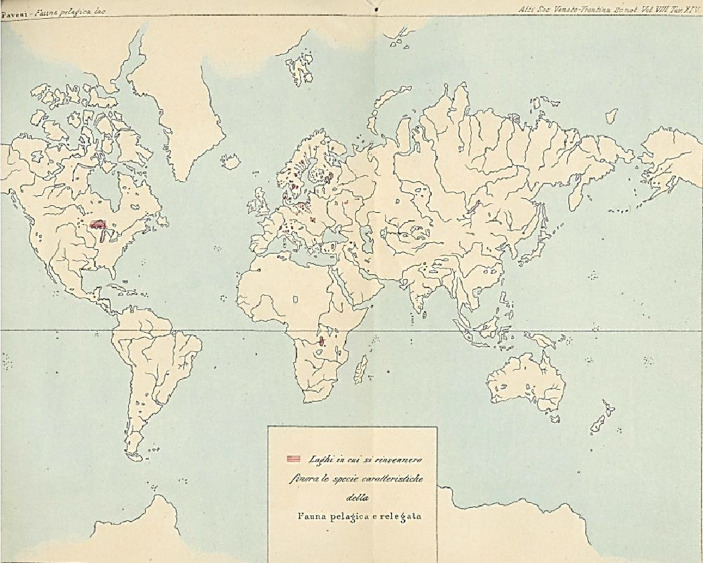
Map showing the lakes where the small pelagic fauna was found up to 1883 (Pavesi 1883)

Small pelagic fauna had been documented in several localities. Pioneering research on these microorganisms emerged in Scandinavia in the 1850s and 1860s. Wilhelm Lilljeborg (1853, 1862) recorded discoveries in the region, followed by Georg Ossian Sars’s (1867) exploration of Norwegian lakes and Sven Lovén’s (1861) work in Sweden. A significant portion of this work involved the identification and cataloging of species, with Lovén (1861) making some attempts to address phylogenetic questions. However, apart from drawing parallels between freshwater and saltwater crustacean species, Lovén did not delve deeply into the issue. Müller (1867) encountered the fauna in Denmark, whereas Edward Schôdler (1866) described daphnids on the Baltic coast. Central Europe also saw significant discoveries in this regard. Antonín Frič (1871) found small pelagic fauna in Bohemian lakes, and famed Darwinian August Weismann (1874) studied the microfauna of Lake Constance, similar to Müller’s work (1870). As mentioned, Forel (1876b, 1879) explored Lake Leman, unknowingly working parallel to Weismann along similar lines. Outside of Europe, Brandt (1879) discovered pelagic microorganisms in Lake Goktschai (Sevan) in the Central Caucasus, and Kessler (1868) documented small pelagic fauna in Lake Onega, Russia. In North America, Sidney Irving Smith described zooplankton from Lake Superior as early as 1871, and wrote several articles on freshwater crustaceans, such as copepods, but did not delve into the phylogeny of small pelagic species (Smith 1871, 1886). Finally, Pavesi (1877) initiated research on Italian lakes, providing a map of locations where the same fauna had been recorded in Europe, Africa, and North America (Fig. 6).

In this context, Pavesi and Forel emerged as key figures in a debate on the origin of pelagic species. Pavesi, supported by many Italian limnologists, promoted the hypothesis of a marine origin for the pelagic fauna. In contrast, Forel supported the idea of passive diffusion.

## François Forel’s Hypothesis: Passive Distribution

Forel explained his hypothesis regarding the origin of small pelagic fauna in several works. He introduced the idea of passive diffusion in the journal *Bulletin de la Société Vaudoise des Sciences Naturelles* starting in 1867 (1867, 1877b, 1882a) and explored it in further works (Forel 1882a, b, [Bibr CR42][Bibr CR43]).^16^ According to Forel (1877a, 1882b), to trace the origins of the small pelagic fauna, it is necessary to consider the period starting from the end of the Ice Age. For alpine lakes, and especially for Forel the Swiss Lake Leman, the Ice Age represented an absolute limit that did not allow local species differentiation. Moreover, Forel assumed that the Ice Age represented a truly inviolable barrier, preventing any biological continuity (Forel 1882b). Consequently, Forel argued for repopulation after the Ice Age.

**Fig. 7:**
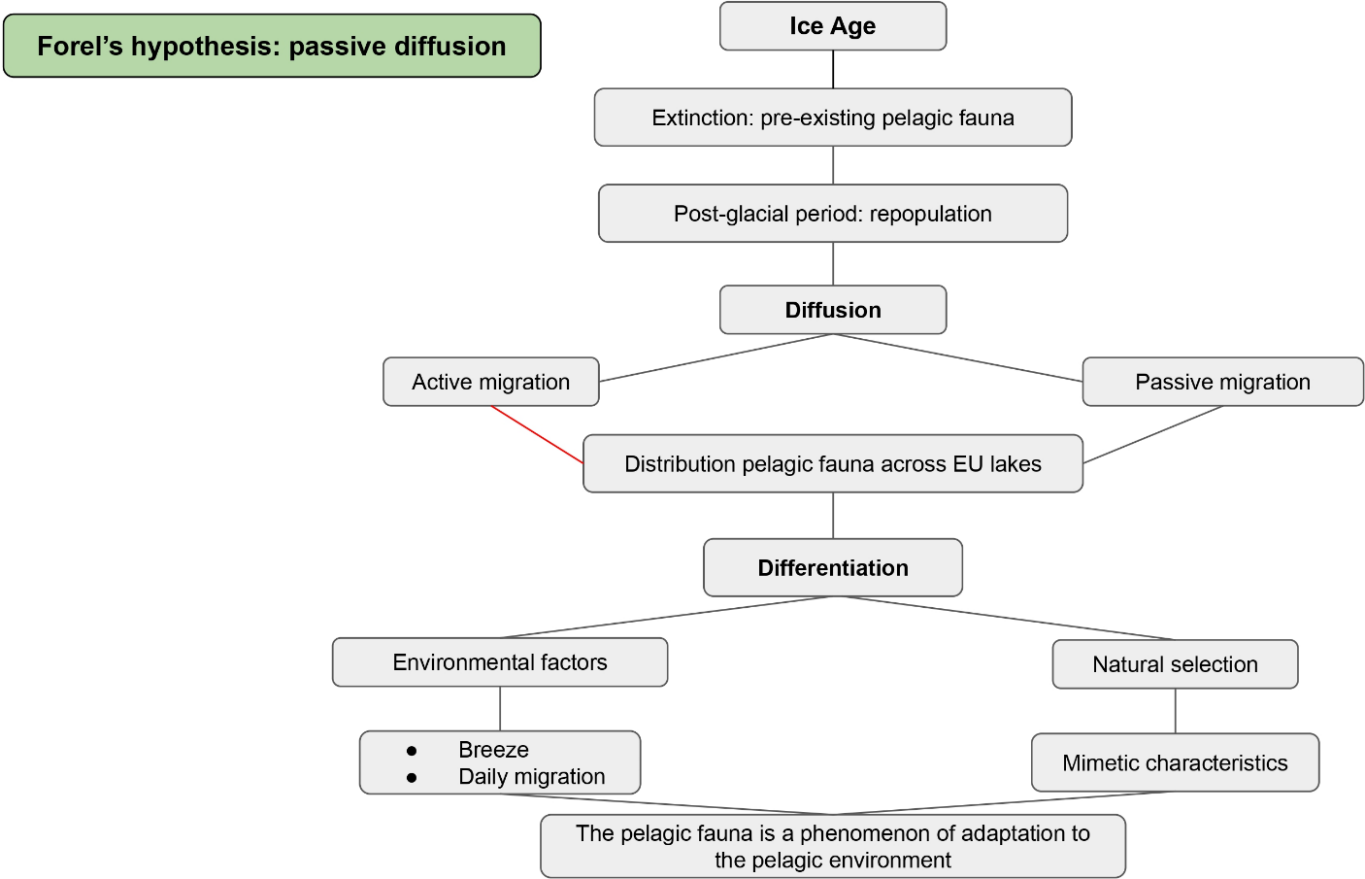
Forel’s hypothesis

Forel started with specific data already gathered to develop his hypothesis of passive diffusion, the transfer of freshwater microfauna from elsewhere to the Alpine lakes (Fig. 7). Indeed, his writing reflects a keen awareness that small pelagic fauna (especially entomostracan) distribution around European lakes is highly uniform (Forel 1882b). He supported his case by pointing to a number of catalogs that had already been published by other scientists, noting how pelagic species are more or less the same in Swiss, Italian, and Scandinavian lakes. Considering the seemingly inarguable biological similarity across a broad geographical area, Forel argued that diffusion and differentiation constituted the likeliest explanation., Forel proposed two possibilities for the mechanism of diffusion: active migration and passive migration. Between the two, he maintained that passive migration was the more important process in the distribution of pelagic fauna across European lakes. According to Forel, “[t]he dissemination of this pelagic fauna is due to passive migration from one lake to another through passing birds; this is proven by the remarkable uniformity of the pelagic fauna in all the lakes of our continent, in the Scandinavian, Swiss lakes, Italian or Caucasian, in the lakes of the subalpine region, or sub-Apennine, in the lakes of the modern or of very ancient origin” (Forel 1885, p. 151, trans. author).^17^

Moreover, Forel argued that to comprehend homogeneity in pelagic species distribution, it is necessary to consider lakes on a broader scale – i.e. European – and not focus on single differences between nearby lakes. These differences should be regarded as a consequence of the contingent nature of the passive diffusion process. According to this account, Forel pointed out that the differentiation of small pelagic species is not a localized process confined to a single lake system. He did not, however, rule out the possibility of differentiation within a single lake. These species, he continued, could be subsequently distributed through passive migration to other lakes. According to Forel, the causes of differentiation – resulting from common environmental factors and natural selection – were not confined to a single lake system. Moreover, he argued that typical biological characteristics of zooplankton, such as transparency, should be interpreted as adaptive mimetic features obtained through the process of natural selection. Forel wrote:

[A] differentiation takes place by natural selection, until at last, after a certain number of generations, there remain only the wonderfully transparent and almost exclusively swimming animals that we know. When this differentiation has taken place, the pelagic species is conveyed by migratory water-birds from one country to another and from one lake to another, where it reproduces its kind if the conditions of existence of the medium are favorable. In this way we may find the pelagic Entomostraca in lakes which are too small to possess the alternation of winds, the animals having been differentiated by the action of the winds in other, larger lakes. (Forel 1882c, pp. 324–325)

Forel assumed that the existence of small pelagic fauna represented a phenomenon of adaptation to the pelagic environment, a process that occurs through passive distribution and differentiation driven by environmental factors and natural selection.

## Pietro Pavesi’s Hypothesis: Marine Origin

**Fig. 8:**
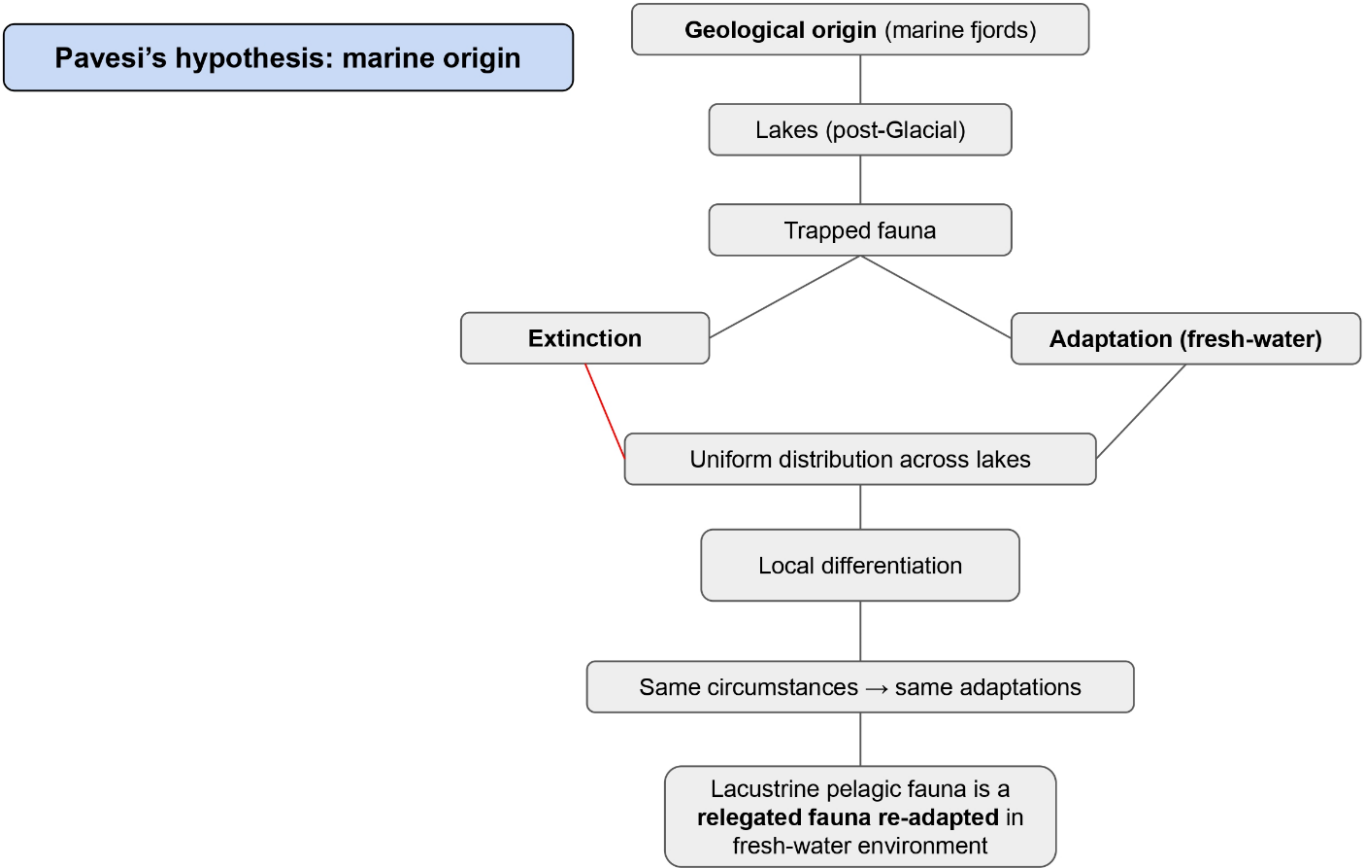
Pavesi’s hypothesis

Pavesi held to an alternative view. He discussed his hypothesis of the marine origin of small pelagic fauna in three works (Pavesi 1879b, 1880, 1883). He summarized his hypothesis as follows: “I think that while our fiords were converted into lakes or the sea left pools within the moraine circle of glaciers, a marine fauna remained imprisoned there. This fauna, becoming lacustrine, did not die out entirely in its lowest representatives of the zoological series” (Pavesi 1879b, p. 706, trans. author).^18^ Pavesi’s hypothesis was an application of the geological and geographical corollaries to animal life. In other words, Pavesi invoked geological studies regarding the origins of lakes and drew on chorological research on the spatial distribution of species, to explain the issue of the origin of small pelagic fauna. He believed that northern Italian lakes, particularly those located in the Insubric region such as Maggiore, Como, Lugano, Orta, were marginal fjords of the Pliocene Sea, barred by moraines and isolated by land uplift. In this context, the lake was reduced to an arm of the sea, and its population consisted of marine fauna relegated and separated from its fauna of origin, with physiological adaptation to fresh water followed by evolution in situ towards lacustrine fauna. While Pavesi assumed the possibility of the distribution of small pelagic fauna through passive diffusion 1879b), he excluded this modality for the subalpine lakes (Pavesi 1883) (Fig. 8).

According to Pavesi, the sea was the center of origin of the small pelagic fauna. Furthermore, this hypothesis posited a biological continuity before and after the Ice Age. He contended that these fjordic lakes, when glaciers invaded them, must have been liquid at the bottom because of ablation, leaving behind residues of their fauna. This challenged the prevailing view of the lifeless Ice Age, and built on the work of Stoppani, who argued for the glacial origin of northern Italian lakes. Pavesi linked these lakes to the classic marine fjords of Scandinavia. Pavesi’s core argument was that the distribution and uniformity of these species across European lakes could be explained by their shared marine origin. He noted the presence of pelagic microorganisms in some Italian lakes such as Maggiore, Como, and Lugano, but not in others such as Trasimeno and Mantua. For Pavesi, this distribution hinged on a lake’s geological origins. Only lakes with marine origin would harbor pelagic species.

Based on these observations, Pavesi built a general hypothesis to the effect that “typical forms of pelagic fauna exist in a very large number of lakes around the globe because they were left there by the sea during the Ice Age” (Pavesi 1883, p. 67, trans. author).^19^ Some of the marine fauna trapped in fjords became extinct, while others adapted to the freshwater environment. Evidence already existed for such adaptation from saltwater to freshwater. Moreover, Pavesi argued that characteristics of pelagic crustaceans (entomostracans) arose from this adaptation and the constant struggle for existence in their new freshwater environment. He wrote:

We know that the pelagic fauna of lakes, of particular interest for adaptation and phylogenesis, is made up of small Crustacea (Entomostracae), differing greatly in form and lifestyle from coastal and lake-bottom Crustacea. These pelagic Entomostracans are transparent and equipped with swimming appendages, an adaptation to the surrounding environment and the struggle for existence. Like swallows and seagulls in the air, these little crustaceans swim without pause or rest in the water for the duration of their lives.^20^ (Pavesi 1880, p. 152, trans. author)

Pavesi further argued for local differentiation within these lakes. He reasoned that the similarity of pelagic species across European lakes, and their relatively uniform distribution, stemmed from a fundamental principle: similar environments select for similar evolutionary traits, leading to what we now call convergent evolution. He wrote “[t]he pelagic fauna of the Ticino and Italian lakes is the same as that of lakes in other European and American countries, confirming a law of adaptation that could be expressed as follows: identical environments require the same adaptations of form” (Pavesi 1880, p. 156, trans. author).^21^ Within this Darwinian framework, therefore, Pavesi traced a marine origin for the small pelagic fauna. He accepted a continuity between the periods before and after the Ice Age and linked the geological origins of the lakes to the presence of small pelagic fauna in contemporary lakes. He advocated for local differentiation through evolution under similar selection pressures, resulting in the uniform distribution of species in similar environmental conditions. Furthermore, by attributing a marine origin to pelagic microorganisms, Pavesi sought a theory based on regular causes, avoiding explanations rooted in accidental events.

## Convergences and Divergences Between Pietro Pavesi and François Forel

Thus the debate on small pelagic fauna was a biogeographic issue framed within an evolutionary context. The main concern was clarifying species’ origin and distribution in lake environments. This perspective involved a biogeographic dimension that emerged early in limnological studies. Forel and Pavesi analyzed the presence of plankton in lakes, focusing particularly on their origin and distribution. Their theories also addressed the adaptation of species to new environments, embracing the concept of evolution through Darwinian processes.

Before Pavesi, the idea of a marine origin for small pelagic fauna had already emerged in Scandinavian countries, particularly through the ideas of Lovén. Certain pelagic crustaceans had previously been documented exclusively in marine environments, and Lovén attributed their presence in Scandinavian lakes to the Ice Age, suggesting a marine origin (Lovén 1861). However, the precise mechanism behind their presence remained unclear. In contrast, Weismann, who had extensively studied small pelagic fauna in Lake Constance, endorsed Forel’s theory of passive diffusion. He deemed the marine origin hypothesis impossible because of the complete discontinuity introduced by glaciation (Weismann 1877, p. 24).^22^

Forel and Pavesi began with the same basic fact: that pelagic fauna, with roughly similar species and varying concentrations (higher in Scandinavia and lower in northern Italy), were found across Europe. From these premises, they proposed contrasting accounts to explain the phylogenesis of these species. Pavesi favored a marine origin, while Forel suggested passive distribution by biological means. Both scientists accepted the validity of evolution by natural selection. For Forel, the Ice Age represented an absolute barrier to species continuity. Pavesi challenged this dogmatic assumption. His hypothesis for the origin of pelagic fauna in lakes, he pointed out, required – and if borne out, proved – that the Ice Age was not “a period of death” (Pavesi 1883). Pavesi rejected Forel’s hypothesis in large part due to concern about its implications. Indeed, Forel’s explanation would have forced Pavesi to assume one or more lakes as the original centers of dispersal that gave rise to the pelagic species. This, in turn, would have forced him to address the difficult question of how far back in geological time such original centers could be placed.

As noted, Pavesi did not accept the idea of lakes as centers of diffusion for the pelagic fauna of Europe. Here, their views diverge significantly. Forel believed that the differentiation process was decentralized, with species diversifying in different lakes and then spreading passively, explaining the uniformity of species on a large geographic scale. In contrast, Pavesi saw localized differentiation along parallel lines, leading to a relatively uniform distribution, as a logical consequence of his preferred hypothesis: a relegated fauna undergoing an evolutionary process in situ.^23^ Furthermore, Pavesi observed irregularities in species distribution on a local scale, with nearby lakes displaying clear differences. This evidence functioned as an argument in favor of passive distribution for Forel, and against it for Pavesi. Forel believed that such irregularities reflected the accidental nature of passive distribution. Pavesi, meanwhile, argued that neighboring lakes should not exhibit irregularities since distribution should be straightforward, and that instead these differences reflected the marine or non-marine origins of the lakes.

According to Forel, if passive distribution is accepted, pelagic species are not forced to undergo local differentiation and the irregularity of distribution can be attributed to its contingent nature. Pavesi, however, regarded this idea as a simplistic solution to a more complex biogeographical question (Pavesi 1883). Contemporary Darwinians such as Hillyer Giglioli felt that the concept of passive distribution, even as articulated by Darwin regarding island populations, represented an extreme recourse that should be limited to explaining cases where no other solution can be found (Giglioli 1874). Pavesi followed Giglioli, and moved away from a Darwinian solution in biogeographic terms. Instead, Forel’s hypothesis fit with Darwin’s own biogeographic ideas and the dispersalist viewpoint (Mayr 1982; Bueno-Hernández et al. 2023).

In principle, Pavesi’s idea of a marine origin and Forel’s passive distribution could coexist as explanations of different aspects of the small pelagic fauna. Forel even conceded a marine origin for *Leptodora hyalina* and *Bylholrephes longimanus*, while rejecting it as a general theory. Pavesi initially considered passive diffusion as complementing his marine origin theory, although later considered them incompatible (Pavesi 1883). Thus, the debate reveals that these two explanations need not be mutually exclusive; while they may have differed in detail, an account of marine origin could potentially include passive distribution. For instance, a marine origin could be reconciled with the possibility of passive distribution within Pavesi’s framework, where marine species adapted to its new freshwater environment and then continued to spread passively.

The late 19th and early 20th centuries saw renewed efforts to clarify the origin of the small pelagic fauna. Monti (1929) briefly discussed the subject. Garbini (1894) authored one of the first comprehensive reports on Lake Garda. He challenged Pavesi’s marine origin hypothesis, favoring Forel’s explanation of passive distribution. Garbini presented two main arguments against Pavesi’s ideas. First, he contested the assertion of a marine origin for Lake Garda, challenging the geological determinism that, for Pavesi, would drive the evolution and distribution of small pelagic fauna. Garbini also questioned the proposed mechanisms for the preservation of these species in Italian lakes during glacial periods. He argued that existing geological data contradicted the idea of a liquid lake bottom beneath glaciers that could have allowed the survival of marine fauna (Sacco 1885). Essentially, Garbini believed that Pavesi’s hypothesis, to be valid, required pelagic fauna today to be found exclusively in fjord lakes and absent in, for instance, high alpine lakes. Garbini instead adopted passive distribution, likely originating from Scandinavian countries: “The secondary limnetic faunas of the various continents all originated by passive immigration from Nordic centers; and the European ones in particular, had their common and unique dispersive center in the lakes of the Scandinavian region, of which the typical limnetic faunas had their cradle in the Baltic fjords, and their origin in the North Sea” (Garbini 1894, p. 31, trans. author).^24^

For Garbini, certain lakes (generally northern) had separated from the sea and hosted a fauna that became lacustrine. Other lakes, lacking a marine connection, acquired their pelagic populations through passive migration. This perspective remained the dominant view in the scientific community. Baldi (1950) revisited the topic, acknowledging Garbini’s critiques of Pavesi’s multiple marine origin hypothesis. He focused on the importance of scientific inquiry in challenging established assumptions, both by evaluating past findings and exploring new research directions. As for Pavesi’s results, they were primarily refuted on a geological basis, as the marine origin of the Insubrian lakes was not confirmed over time. The idea of post-Ice Age repopulation via species migration systems offered the most promising direction for new research and results.

## Conclusion

The topic of pelagic fauna has attracted the attention of scholars worldwide since the second half of the 19th century, coinciding with the formalization of limnology as a scientific discipline (Egerton 2014). This essay has traced the discovery of plankton in lake systems and the ensuing debate on plankton phylogeny, particularly within the Swiss and northern Italian contexts, as explored by Forel (1882b, 1885) and Pavesi (1883). Their debate revolved around four main topics: the impact of the Ice Age; the significance of a new trophic level in lakes (zooplankton), and its contribution to understanding of nutrient cycling and the concept of the lake as a *microcosm*; differing biogeographic theories about species distribution and movements among lakes; and Darwinian concepts of evolution and adaptation through natural selection. These subject areas shaped the beginning of limnology as a discipline, leading to its formal establishment (Elster 1974). In the debate between Pavesi and Forel, these topics intersected and the resolution involved the application of Darwinian evolutionary ideas, biogeographical evidence, and a richer understanding of nutrient cycling and trophic chains.

While both Forel’s and Pavesi’s perspectives considered the evolution of species, adopting distinctly Darwinian views, they offered different perspectives on the Ice Age – for Forel, it was a biological barrier, and Pavesi saw continuity in species between the periods before and after. However, their main focus was on the origin and distribution of plankton species in lakes, how these species settled in particular territories, and the existence of a homeland of origin. These were the questions that Pavesi, Forel, and other early limnologists faced when confronted with this limnological biogeographical problem.

The beginning of limnology was, at least in part, rooted in limnological biogeography. Contemporary literature highlights a close relationship between ecology and biogeography, both historically (McIntosh 1985) and conceptually (Jenkins and Ricklefs 2011). The biogeographical dimension of terrestrial ecological disciplines at their origins has been pointed out (Egerton 2019). I have argued that the discovery of small pelagic plankton and the subsequent debate similarly shed light on the biogeographical origins of limnology, which historical treatments have so far largely overlooked (Acot 1998). The dispute between Pavesi and Forel was, after all, partly biogeographical in that it centered on the processes that gave rise to the current distribution of lake zooplankton species.

The discovery of plankton in lakes marked a pivotal moment in the development of limnology as a scientific discipline (Acot et al. 1998). According to Forel (1888), it provided the *missing link* in understanding the cycle of organic matter in lake systems. Together with Bertola (1998), I argue that the discovery of plankton in lake systems brought further conviction to the idea that lakes were indeed *microcosms*, echoing Forbes’s earlier ideas (1887) and anticipating those later expressed by Forel (1892–1904).^25^ Thus the study of plankton in lakes should be historically recognized as crucial to identifying the mutual trophic relationships among organisms and the relationship between organisms and the environment. These are fundamental assumptions underlying the ecosystem concept (Hagen 1992; Golley 1993).

Finally, this essay has contributed to the literature on the origins of the Italian limnological tradition, which has attracted some recent historical attention. For instance, a study on Emilio Corti’s bibliography includes a comparative analysis of limnological research topics between the past and the present (Mosello et al. 2012). The Water Research Institute (CNR) in Verbania-Pallanza has been emphasized as a key institution and ist archives discussed as a valuable source of documentation (Mosello 2021). Some scholarship on the figure of Rina Monti has also been produced (Dröscher 2007; Mosello and Fontaneto 2017). This essay has emphasized the significance of Pavesi and his biological work, and the importance of northern Italy more generally. It is to be hoped that future research will continue this trend and illuminate the contributions to limnology of scientists outside those regions that have so far gained the most historical attention, northern Europe and the United States.

## Footnotes

1 Pavesi and Forel held differing views on the impact of the last Pleistocene ice age on species continuity. Forel regarded it as an absolute barrier, arguing that it prevented small pelagic animals from surviving the transition. For Forel, “in the case of our Swiss lakes the glacial period forms an absolute limit, which entirely prevents our accepting a local differentiation of the old Tertiary species and their transformation into the existing species” (Forel 1882c, p. 324). In contrast, Pavesi believed that this barrier did not exist, maintaining that there was biological continuity before and after the Ice Age. That is, for Pavesi the universality and uniformity of fauna implied the species were relicts (Pavesi 1883, p. 65).

2 For more on Kircher, see Reilly (1955) and Findlen (2004). For more on Marsili see Sciarra (2012).

3 See Ricciardi (1925b) for a comprehensive historical review of the early Italian limnological tradition focusing especially on geography. During the first decades of the 20th century, he conducted limnological research of his own (Ricciardi 1925a).

4 See also Ramsay (1862) and Gabriel de Mortillet, and Pigorini (1898) for a bibliography of Mortillet’s work in Italy.

5 Stoppani described his hypothesis on the origin of Alpine lakes as follows: “The Lombardy lakes are nothing more than arms of the sea or fjords, occupied by glaciers during their advance, blocked downstream, or towards the sea by moraine reliefs and therefore intercepted in such a way that, remaining empty when the glaciers retreated, they necessarily had to be converted into freshwater lake” (Stoppani 1879, p. 306, trans. author). [I laghi lombardi non sono altro che bracci di mare o fjords, occupati dai ghiacciai durante il loro avanzamento, sbarrati a valle, ossia verso mare dai rilievi morenici e perciò intercettati in tal guisa che, rimasti vuoti al ritirarsi dei ghiacciai, dovettero necessariamente convertirsi in laghi d’acqua dolce].

6 After discussing Forel’s hypothesis on passive diffusion, Stoppani introduced Pavesi’s work as follows: “Professor Pavesi […] wonders if a more satisfactory solution to the problem is not offered by the theory of the formation of subalpine lakes proposed by Stoppani […]. The marine creatures, […] would be nothing more than the descendants of those that populated the same sea arms or fjords when, prior to the invasion of the glaciers, the sea freely extended into the Alps” (Stoppani 1879, p. 306, trans. author). [Il Prof. Pavesi […] si domanda se una soluzione più soddisfacente del problema non sia offerta dalla teoria della formazione dei laghi subalpini che l’ab. Stoppani sostiene […]. Gli animaletti marini […] non sarebbero che i discendenti di quelli che popolavano gli stessi bracci di mare o fjords quando, anteriormente all’invasione de’ ghiacciai, il mare penetrava liberamente in seno alle Alpi].

7 For a recent account on the figure of Leopoldo Maggi and his scientific historical importance, see Brömer (2011).

8 Original Italian text: L’intento primo di questo mio esame, era di conoscere quali forme di Protisti le acque dei nostri laghi contenessero; eppero, dopo le ricerche del chiarissimo Prof. Pietro Pavesi, fatte con buon successo, intorno alla loro fauna pelagica, mi si presentava, un tale esame, interessante anche sotto questo punto di vista. He explored dinoflagellates and the genus *Ceratium* (Maggi 1880a, c). Moreover, Maggi delved into the technical issues of Pavesi’s hypothesis on the origin of pelagic fauna, drawing a connection between the importance of geology in the study of plankton phylogeny (see Maggi 1880b, p. 44).

9 Figure 3 shows only six of the 32 lakes that have been analyzed by Pavesi (1883); these were Avigliana, Ritom, Mantova, Ledro, Loppio, Caldonazzo, Levico, Revine, Santa Croce, Alleghe, Lugano, Maggiore, Como, Garda, Trasimeno, Albano, Idro, Varese, Comabbio, Monate, Ghirla, Mergozzo, Orta, Lupero, Iseo, Annone, Spinone, Azegli, Biandronno, Monate, Alberio, and Viverone.

10 For a thematical biographical account, see Bertola (2012). On Forel’s scientific legacy, see (Girardclos et al. 2012). On the history of the Forel Institute, see Dominik and Loizeau (2012), and Slaveykova (2012). Moreover, Bertola (2012) briefly pointed out Forel’s discovery of deep and pelagic fauna in Lake Geneva and his interest in zooplankton populations and their nocturnal and diurnal migrations. He also discussed Forel’s research on microbes, arguing for the first recognition of a lake as a heterotrophic ecosystem (Vincent and Bertola 2012). On the importance of the concept of the circulation of organic matter, also see Acot et al. (1998), in particular concerning Forel’s (1901) work on Lake Leman, although Acot et al. do not specifically discuss pelagic fauna.

11 Egerton (2014, 2015) further contextualized Forel’s role in the development of limnology.

12 See Balvay (1984), for an account of “entomostracan” crustaceans at Lake Leman.

13 See also Forel (1877a) for an account on pelagic flora.

14 Original French text: Ces découvertes sont précieuses, elles nous donnent les chaînons qui nous manquaient pour comprendre dans nos lacs le cycle de la circulation de la matière organique, entre la nature morte et la nature vivante.

15 Although several terms, such as “microcosm,” are considered precursors to the ecosystem concept, the term “ecosystem” was formally introduced by Tansley in 1935 and later developed by Lindeman (1942) in his limnological research. For a historical discussion on the ecosystem concept, see Hagen (1992), Golley (1993), and Jax (1998).

16 For a bibliographical account regarding Swiss lakes, see Walser and Collett (1913).

17 Original French text: Mais la dissémination de cette faune pélagique est due à la migration passive d’un lac à l’autre par le moyen des oiseaux de passage; cela est prouvé par l’uniformité remarquable de la faune pélagique dans tous les lacs de notre continent, dans les lacs scandinaves, suisses, italiens ou caucasiques, dans les lacs de région subalpine, ou subapennine, dans les lacs d’origine moderne ou d’origine très ancienne.

18 Original Italian text: Così dunque io penso che, mentre i nostri fiords si convertirono in laghi od il mare lasciò delle pozze entro la cerchia morenica dei ghiacciai, siavi rimasta imprigionata una fauna marina, la quale, divenendo lacustre, non si spense del tutto ne’ suoi rappresentanti più bassi della serie zoologica.

19 Original Italian text: Abbiamo elementi fortissimi per credere che le forme tipiche della fauna pelagica esistono in un grandissimo numero di laghi del globo perché vi furono abbandonate dal mare durante l’epoca glaciale.

20 Original French text: On sait que la faune pélagique des lacs, d’un intérêt tout spécial en vue de l’adaptation et de la phylogenèse, se compose de petits Crustacés (Entomostracés), différant beaucoup par les formes et le genre de vie des Crustacés côtiers et du fond des lacs. Par adaptation au milieu ambiant, en vue de la lutte pour l’existence, le corps de ces Entomostracés pélagiques est transparent et muni d’appendices propres à la nage. Comme les hirondelles et les mouettes dans les airs, ces petits Crustacés nagent sans trêve ni repos dans les eaux pendant tout le temps de leur vie.

21 Original French text: La faune pélagique des lacs tessinois et italiens se trouve être la même que celle des lacs des autres pays d’Europe et d’Amérique; fait qui confirme une loi d’adaptation que l’on pourrait exprimer comme suit: des milieux identiques exigent les mêmes adaptations de formes.

22 In one of his writings on pelagic fauna, Weismann (1874) devoted a comprehensive monograph to *Leptodora*. In a subsequent work, based on a talk in Freiburg during the winter of 1875-76, he expanded his focus to freshwater organisms, presenting their complex ecosystems rather than merely listing species. His research at Lake Constance highlighted animal communities in freshwater systems and delved into broader limnological issues (Straile 2021). He discussed the diurnal migration of plankton, its implications, and the depths to which these organisms descend. Addressing the origin of pelagic fauna, Weismann supported Forel’s idea of passive distribution. For instance, Weissmann (1877) said “[a]ll these animals must have migrated from other lakes, in which they have been developed from ancient times, but not as living individuals, only as seeds!” (Weissmann 1877, p. 25, trans. Author). Weismann noted the difficulties of active transport for small pelagic species. He questioned how Leptodora could ascend the Rhine or survive in streams, suggesting passive egg transport as the key to their wide distribution in European lakes (Weismann 1877, p. 27). Weismann’s link to Darwin influenced his advocacy for passive distribution and highlighted structural changes in organisms once they settled in different environments (Mayr 1985).

23 In his 1880 French paper, Pavesi supports the idea that identical environments lead to identical adaptations (circonstances identiques exigent les mêmes adaptations). He argues that marine species isolated in different lakes “adapt” to fresh water in the same way. This evolutionary view may be rooted in a Lamarckian influence, differing from the more strictly Darwinian perspective found in Forel. Pavesi’s position is informed by his views on geology, particularly the role geological factors play in the distribution of species across specific geographic areas. He suggests that these species then evolved similarly due to similar environmental pressures, implying that the same ancestral species underwent comparable evolutionary changes, resulting in the same species appearing in different lakes. The Lamarckian view emerges according to the principle that similar selection pressures would necessarily produce the same species, bringing out the direct influence of their environment (lacustrine). Nevertheless, like Forel, Pavesi acknowledged that some species do not differ significantly from their marine ancestors and does not dismiss the possibility of species diverging from their founders. For instance, he cites Thomas H. Huxley from *The Crayfish* (1880, p. 130), noting that “in all cases, it appears reasonable to apply the analogy of the *Mysis relicta* and to suppose that the fluviatile prawns are simply the result of the adaptive modification of species which, like their congeners, were primitively marine.”

24 Original Italian text: Le faune limnetiche secondarie dei vari continenti ebbero tutte origine per immigrazione passiva da centri Nordici; e quelle Europee in particolare, ebbero il loro centro dispersivo comune ed unico nei laghi della regione Scandinava, di cui le faune limnetiche tipiche ebbero loro culla nei fiordi Baltici, e loro origine nel Mare del Nord.

25 See also Jenkins (2014) for a recent account on the concept of microcosm.
